# An inverted J-shaped association of serum uric acid with muscle strength among Japanese adult men: a cross-sectional study

**DOI:** 10.1186/1471-2474-14-258

**Published:** 2013-08-30

**Authors:** Cong Huang, Kaijun Niu, Yoritoshi Kobayashi, Lei Guan, Haruki Momma, Yufei Cui, Masahiro Chujo, Atsushi Otomo, Hui Guo, Hiroko Tadaura, Ryoichi Nagatomi

**Affiliations:** 1 Department of Medicine and Science in Sports and Exercise, Tohoku University Graduate School of Medicine, Sendai 980-8575, Japan; 2 Department of Epidemiology, School of Public Health, Tianjin Medical University, 22 Qixiangtai Road, Heping District, Tianjin 300070, China; 3 Division of Biomedical Engineering for Health and Welfare, Tohoku University Graduate School of Biomedical Engineering, 2-1 Seiryo-machi, Aoba-ku Sendai 980-8575, Japan

**Keywords:** Grip strength, Leg extension power, Antioxidant, Reactive oxygen species, Inflammation

## Abstract

**Background:**

Uric acid (UA) may protect muscle function from oxidative damage due to reactive oxygen species through its powerful antioxidant capacity. However, several studies have demonstrated that hyperuricemia is closely related to systemic inflammation and has oxidant properties effects, both of which may increase the risk of muscle strength loss. The purpose of this study was to examine the association of serum UA concentration with grip strength and leg extension power in adult men.

**Methods:**

This study is a cross-sectional survey in which 630 Japanese male employees aged 30 years and older participated. Five hundred and eighty-six subjects participated in the measurement of grip strength, and 355 subjects participated in the measurement of leg extension power. Blood samples were obtained for serum UA analysis.

**Results:**

After adjustment for potential confounders, grip strength differed significantly between participants with and those without hyperuricemia (geometric mean and 95% confidence interval [CI]: 40.3 [39.2–41.3] kg vs. 41.9 [41.3–42.5] kg; *P* = 0.01). In addition, serum UA levels (quartiles) showed an inverted J-shaped curve with grip strength (mean and 95% CI: Q1, 41.6 [40.6–42.6] kg; Q2, 42.2 [41.2–43.2] kg; Q3, 41.8 [40.8–42.8] kg; Q4, 40.4 [39.3–41.4] kg; *P* for quadratic trend = 0.05). The results in the leg extension power group were similar to those observed in the grip strength group.

**Conclusion:**

This population-based cross-sectional study shows for the first time that hyperuricemia is associated with poor muscle strength. Moreover, the results indicate an inverted J-shaped association between serum UA quartiles and muscle strength.

## Background

Sarcopenia is defined as a gradual decline in skeletal muscle mass with aging and is associated with poor muscle strength [1]. Increasing evidence has demonstrated that low muscle strength is a strong and consistent predictor of mortality in middle-aged and elderly persons [2-4]. One important contributing factor to the development of sarcopenia is postulated to be the accumulation of reactive oxygen species (ROS), which may cause the oxidative damage of protein and DNA in skeletal muscle [5].

Numerous antioxidants circulating in blood or present in tissues may help reduce oxidative stress; however, of these, uric acid (UA) has long been thought to be one of the most important compounds involved in ROS removal. Maxwell et al. [6] have implied that UA probably accounts for more than half of the free radical–scavenging capacity in serum. Specifically, UA can scavenge oxidants such as hydroxyl and peroxyl radicals and singlet oxygen [7,8], and can prevent the generation of radicals induced by peroxynitrite decomposition [9]. Recently, a 3-year follow-up study [10] reported that UA could positively influence skeletal muscle strength in the elderly, probably by providing protection against oxidative stress.

In contrast to its role as an antioxidant, high concentrations of UA have also been considered a trigger for a number of diseases. Hyperuricemia, usually defined as a UA concentration of >7.0 mg/dL in men and >6.0 mg/dL in women [11], is a risk factor for cardiovascular disease, hypertension, diabetes, and kidney disease [12]. Additionally, serum UA might contribute to the inflammatory response and the subsequent inflammatory related diseases [13,14].

On the basis of studies conducted during the last 3 decades, it is difficult to define UA as clearly beneficial [7,10,15] or pathological [12]. Ruggiero et al. [16] reported that the elderly in the middle serum UA quintile tended to have less disability in instrumental activities of daily living and higher physical performance as measured by walking speed, standing balance and ability to rise from a chair, than those with higher or lower UA levels. In fact, the antioxidant compound UA can become a pro-oxidant in specific environments, particularly when present in blood at higher than normal levels [17].

In light of these observations, we hypothesize that low or high serum UA concentration may be associated with poor skeletal muscle strength. Therefore, the aim of the present study was to investigate the association between serum UA and muscle strength, i.e., grip strength and leg extension power, in adult men.

## Methods

### Study population

This cross-sectional analysis was performed as part of the Oroshisho Study, a study of lifestyle-related effects on illnesses and health status in Japanese adults. In this study, all of the 1263 participants were adult employees and received an annual health examination at the Sendai Oroshisho Center in Sendai, Japan, 2009. This study was carried out from August 3 to 7. In addition to the annual health examination, physical activities, daily nutrient intakes, and muscle strength were also assessed. Further details on the study have been provided elsewhere [18,19].

The analysis in current study was limited to participants who agreed to provide written informed consent for their data to be analyzed (n = 1215, response rate = 96.2%). Subjects were excluded from the present study for the following reasons: 1) being female (n = 282); 2) having no UA data available (n = 136); 3) having a history of cardiovascular disease and renal failure, or using antihypertensive, lipid-lowering, or antidiabetic agents (n = 146) because of the concern that such diagnoses and agents may lead to changes in UA levels and/or muscular function and thus confound and obscure a true association [19]; or 4) having no data on education level, physical activity, depression symptoms, and diet (n = 21). None of the participants had gout. With this population sample, subjects with missing data on grip strength (n = 44) or were not measured for leg extension power (n = 275) were excluded from analyses. Finally, a total of 586 subjects qualified for grip strength analysis and 355 subjects qualified for leg extension power analysis. All research procedures were consistent with the Declaration of Helsinki for human rights [20]. Approval for this study was obtained from the institutional review board of the Tohoku University Graduate School of Medicine.

### Measurement of serum UA

Blood samples were drawn from the antecubital vein in the morning, after overnight fasting, while subjects were in the sitting position; blood was collected into siliconized vacuum glass tubes; and then the frozen serum was sent to Bio Medical Laboratories (BML Inc., Sendai, Japan) for subsequent measurement of blood components. Serum UA levels were measured enzymatically by using a Pureauto S UA kit (Sekisui Medical Co., Ltd., Tokyo, Japan); the lower limit of detection was 0.2 mg/dL. UA levels were then divided into quartiles based on the distribution: <5.4, 5.4–6.0, 6.1–6.8, and >6.8 mg/dL. Hyperuricemia was defined as a serum UA level of ≥7.0 mg/dL for men [11].

### Measurements of muscle strength

Grip strength in kilograms was assessed using a handheld digital Smedley dynamometer (TKK 5401; Takei Scientific Instruments Co., Ltd, Niigata, Japan) in the standing position. Participants were asked to hold the dynamometer in a comfortable position and were not allowed to see the figure displayed on the dial during the trial. Grip strength was measured 4 times, twice for each hand alternately, with a brief interval between trials. Participants were encouraged to exert their maximal grip, and the average value of the highest power of both hands was recorded as the grip strength.

Leg extension power was determined with an isotonic apparatus (Anaeropress 3500; Combi Co., Tokyo, Japan). After warming up, the subjects sat back on the seat, placed both feet on the sliding foot plate with the knee angle adjusted to 90°, and then, the waist of each participant was firmly fastened with belts. Participants were urged to extend their feet with maximum effort. Five trials were measured at 15-s intervals, and the maximal value was recorded as the leg extension power (W/kg) for the analysis. The reliability and validity of the leg extension power measurement was described in detail elsewhere [21].

### Relevant covariates

Body mass index (BMI) was calculated as weight/height^2^ (kg/m^2^). Blood pressure was measured twice on the right arm with an automatic device (Yamasu 605P; Kenzmedico, Saitama, Japan) while subjects were in the sitting position, with a 5-min interval between measurements. The mean of 2 measurements was used for the current study. Information on age, sex, smoking status (never, former, current), drinking frequency (every day, sometimes, never), education levels (<college or ≥ college), and occupation (desk-centric or not) were obtained using a questionnaire survey.

The estimation of physical activity was determined using the International Physical Activity Questionnaire [22]. Total daily physical activities were calculated [22] and divided into 2 categories, <23 and ≥23 metabolic equivalent (MET) hours/week [23]. Assessment of daily nutrient intake was made using a brief self-administered diet history questionnaire (BDHQ) containing questions about the consumption frequency of 75 principal foods [24]. The reproducibility and validity of the BDHQ used in this study were described in detail elsewhere [24]. The metabolic syndrome was defined according to the criteria of the American Heart Association Scientific Statements of 2009 for persons of Asian ethnicity (including Japanese) [25]. The Japanese version of the Self-rating Depression Scale was used to examine the severity of depression subjectively [26]. Participants who scored ≥45 raw sum points were considered to have depression [27].

Serum high-sensitivity C-reactive protein (hs-CRP) was measured with N-latex CRP-2 (Siemens Healthcare Japan, Tokyo, Japan). The lower limit of detection was 0.02 mg/L, and the hs-CRP value was considered to be 0.01 mg/L when lower than the detection limit. Other serum parameters were analyzed by enzymatic methods, using appropriate kits for measuring the concentrations of creatinine (Kainos, Tokyo, Japan), triglycerides, high-density lipoprotein cholesterol (HDL-C) (Sekisui Medical, Tokyo, Japan), and fasting blood glucose (Eurotec, Tokyo, Japan). The estimated glomerular filtration rate (eGFR) in men was calculated from the serum creatinine level by using a new Japanese equation [28], in which the glomerular filtration rate (mL·min^-1^·1.73 m^-2^) = 194 × serum creatinine − 1.094 × age − 0.287.

### Statistical analyses

In this study, continuous variables were summarized as geometric mean (95% confidence interval, CI) and categorical variables were summarized as percentages. Except for leg extension power, all continuous variables were log-transformed because of their abnormal distribution and back-transformed for data presentation.

Analysis of covariance (ANCOVA) was conducted to examine age-adjusted participant characteristics according to serum UA levels. By ANCOVA analysis, differences in muscle strength between subjects with and those without hyperuricemia were analyzed after adjusting for potential confounders. The linear or quadratic trends between serum UA quartiles and muscle strength were analyzed by univariate (analysis of variance, ANOVA) and multivariate analysis (ANCOVA). In multivariate analysis, adjustment for age and BMI was performed in model 1. Model 2 was adjusted for lifestyle-related factors, including smoking status, drinking frequency, education levels, occupation, physical activity, daily energy intakes, and protein intakes in addition to the variables in model 1. Model 3 was adjusted for variables in model 2 and health status–related factors such as metabolic syndrome, depressive symptoms, and eGFR (renal function). Finally, model 4 was additionally adjusted for the circulating inflammatory marker hs-CRP. A significance level of *P* < 0.05 was used for 2-sided tests. All tests were performed using IBM SPSS Statistics 19.0 software (IBM SPSS Inc., Chicago, IL, USA).

## Results

### Participant characteristics

The population sample consisted of 586 men for grip strength analysis (mean age [SD]: 46.3 [9.3] years) and 355 men for leg extension power analysis (46.3 [9.5] years). In both populations, the mean (SD) serum UA value was 6.1 (1.2) mg/dL.

The age-adjusted associations between serum UA and participant characteristics in the grip strength group are presented in Table 1. The mean BMI, systolic blood pressure, diastolic blood pressure, proportion of everyday drinkers, serum hs-CRP, and triglyceride were significantly and positively associated with the quartiles of serum UA (*P* for linear trend <0.01, for all). In addition, the prevalence of metabolic syndrome was higher across the quartiles of serum UA (*P* for linear trend <0.01), whereas the proportion of nonsmokers, former smokers, never drinkers, and sometimes drinkers, and the eGFR demonstrated a negative association with serum UA levels (*P* for linear trend: 0.04, 0.01, 0.06, 0.06, and <0.01, respectively). In contrast, the mean age; the proportion of those who had education levels ≥ college, self-reported physical activity ≥23 MET hours/week, and depressive symptoms; the proportion of desk workers; HDL-C; and fasting glucose did not differ significantly among the serum UA quartiles. In addition, none of the nutrients examined demonstrated any statistically significant associations with serum UA levels.

**Table 1:** Age-adjusted participant characteristics according to serum UA levels in grip strength group (n = 586)

	**Serum UA quartiles, mg/dL**				***P ***^**1**^
	**<5.4**	**5.4–6.0**	**6.1–6.8**	**>6.8**	
Participants	152	144	145	145	―
Age, years	46.4 (44.9–47.8) ^2^	45.3 (43.8–46.8)	45.1 (43.6–46.5)	45.0 (43.5–46.4)	0.18
BMI, kg/m^2^	22.7 (22.2–23.1)	22.9 (22.4–23.4)	23.2 (22.7–23.7)	24.8 (24.3–25.4)	<0.01
Systolic blood pressure, mmHg	122 (120–124)	124 (122–126)	128 (126–131)	132 (130–135)	<0.01
Diastolic blood pressure, mmHg	78 (77–80)	79 (77–80)	82 (80–84)	85 (84–87)	<0.01
Education levels (≥college), %	33.6	31.3	37.9	32.4	0.91
Occupation (desk work), %	77.6	78.5	78.6	77.9	0.86
Smoking status					
Never, %	38.2	38.9	35.9	26.9	0.04
Former, %	10.5	7.6	13.1	18.6	0.02
Current, %	51.3	53.5	51.0	54.5	0.75
Drinking frequency					
Every day, %	17.8	32.6	31.0	37.2	<0.01
Sometimes, %	57.9	55.6	53.8	48.3	0.06
Never, %	24.3	11.8	15.2	14.5	0.05
Physical activity, MET hours/week					
≥23, %	37.5	33.3	33.1	37.2	0.94
Metabolic syndrome, %	18.4	13.9	17.9	30.3	<0.01
Depressive symptoms, %	34.9	31.3	28.3	36.6	0.85
Daily nutrient intakes					
Total energy, kcal	1819 (1728–1914)	1815 (1722–1912)	1855 (1761–1954)	1888 (1792–1989)	0.26
Protein, g	61.6 (58.1–65.2)	59.7 (56.3–63.4)	61.1 (57.6–64.8)	62.6 (59.0–66.4)	0.59
Vitamin C, mg	78.7 (71.7–86.4)	77.9 (70.7–85.7)	75.9 (69.0–83.5)	81.5 (74.0–89.6)	0.72
α-tocopherol, mg	6.3 (5.8–6.7)	6.2 (5.8–6.7)	6.4 (6.0–6.9)	6.5 (6.0–7.0)	0.48
β-carotene, μg	1897 (1660–2167)	1842 (1606–2112)	1967 (1716–2254)	2128 (1857–2439)	0.18
Blood characteristics					
High-sensitivity CRP, mg/L	0.33 (0.28–0.39)	0.37 (0.31–0.45)	0.34 (0.29–0.41)	0.55 (0.46–0.65)	<0.01
eGFR, mL·min^-1^·1.73 m^-2^	84.9 (83.1–86.9)	82.9 (81.0–84.8)	82.5 (80.7–84.4)	76.5 (74.8–78.3)	<0.01
Triglyceride, mg/dL	97.1 (88.9–106)	106 (96.3–116)	115 (105–126)	137 (125–150)	<0.01
HDL-C, mg/dL	52.9 (50.8–55.0)	50.9 (49.0–53.0)	52.0 (50.0–54.1)	50.1 (48.1–52.1)	0.16
Fasting glucose, mg/dL	97.1 (95.0–99.1)	94.2 (92.2–96.3)	94.3 (92.3–96.4)	95.0 (93.0–97.1)	0.20

^1^ The linear trends were obtained using ANCOVA for both continuous and categorical variables.

^2^ Variables have been log-transformed and expressed as geometric means (95% confidence interval).

*Abbreviations*: *BMI* body mass index, *CRP* C-reactive protein, *eGFR* estimated glomerular filtration rate, *HDL-C* high-density lipoprotein-cholesterol.

The results of the main characteristics of participants in the leg extension power group were approximately equal to that of the grip strength group, with the exception of HDL-C and the proportion of never drinkers (Table 2). In the leg extension power group, HDL-C was found to be significantly lower across the serum UA quartiles (*P* for linear trend = 0.01), whereas no association between the proportion of never drinkers and UA quartiles were observed.

**Table 2:** Age-adjusted participant characteristics according to serum UA levels in leg extension power group (n = 355)

	**Serum UA quartiles, mg/dL**				***P ***^**1**^
	**<5.4**	**5.4–6.0**	**6.1–6.8**	**>6.8**	
Participants	92	90	85	88	―
Age, years	45.9 (44.1–47.9) ^2^	45.4 (43.6–47.4)	44.9 (43–46.8)	45.3 (43.4–47.3)	0.58
BMI, kg/m^2^	22.8 (22.2–23.4)	22.9 (22.3–23.5)	23.6 (23–24.3)	24.7 (24–25.3)	<0.01
Systolic blood pressure, mmHg	122 (119–125)	122 (119–125)	128 (125–131)	131 (128–134)	<0.01
Diastolic blood pressure, mmHg	78 (76–80)	77 (75–79)	82 (79–84)	83 (81–86)	<0.01
Education levels (≥college), %	34.8	32.2	41.2	33.0	0.90
Occupation (desk work), %	80.4	77.8	75.3	78.4	0.69
Smoking status					
Never, %	43.5	46.7	40.0	29.5	0.04
Former, %	12.0	7.8	15.3	18.2	0.11
Current, %	44.6	45.6	44.7	52.3	0.36
Drinking frequency					
Every day, %	17.4	26.7	28.2	35.2	<0.01
Sometimes, %	58.7	62.2	54.1	46.6	0.05
Never, %	23.9	11.1	17.6	18.2	0.54
Physical activity, MET hours/week					
≥23, %	41.3	30.0	31.8	37.5	0.72
Metabolic syndrome, %	16.3	13.3	22.4	29.5	<0.01
Depressive symptoms, %	37.0	30.0	28.2	35.2	0.78
Daily nutrient intakes					
Total energy, kcal	1889 (1767–2019)	1772 (1657–1896)	1915 (1787–2053)	1947 (1819–2085)	0.27
Protein, g	63.8 (59.2–68.8)	58.6 (54.3–63.2)	63.8 (59.0–69.0)	65.0 (60.2–70.2)	0.42
Vitamin C, mg	84.0 (74.8–94.2)	78.5 (69.9–88.2)	79.8 (70.8–89.9)	88.0 (78.3–99.0)	0.55
α-tocopherol, mg	6.7 (6.1–7.3)	6.2 (5.6–6.8)	6.8 (6.2–7.4)	6.8 (6.2–7.5)	0.44
β-carotene, μg	2051 (1731–2430)	1812 (1527–2151)	2181 (1828–2601)	2224 (1870–2644)	0.27
Blood characteristics					
High-sensitivity CRP, mg/L	0.28 (0.23–0.35)	0.37 (0.29–0.46)	0.32 (0.25–0.40)	0.48 (0.38–0.60)	<0.01
eGFR, mL·min^-1^·1.73 m^-2^	84.1 (81.9–86.4)	82.2 (79.9–84.5)	82.5 (80.2–84.9)	77.2 (75.1–79.4)	<0.01
Triglyceride, mg/dL	91.4 (82.1–102)	109 (98.2–122)	111 (99.3–124)	129 (116–144)	<0.01
HDL-C, mg/dL	54.5 (51.9–57.3)	51.1 (48.6–53.7)	51.7 (49.1–54.4)	49.5 (47.1–52.0)	0.01
Fasting glucose, mg/dL	94.8 (92.8–97.0)	93.7 (91.6–95.8)	94.5 (92.3–96.7)	94.8 (92.6–96.9)	0.90

^1^ The linear trends were obtained using ANCOVA for both continuous and categorical variables.

^2^ Variables have been log-transformed and expressed as geometric means (95% confidence interval).

*Abbreviations*: *BMI* body mass index, *CRP* C-reactive protein, *eGFR* estimated glomerular filtration rate, *HDL-C* high-density lipoprotein-cholesterol.

### Serum UA and muscle strength

**Figure 1:**
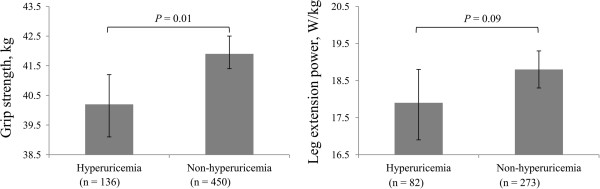
**Adjusted association between hyperuricemia and poor muscle strength.** Adjusted for age, body mass index, education levels, occupation, smoking status, drinking frequency, physical activity, daily energy intakes, daily protein intakes, metabolic syndrome, depressive symptoms, estimated glomerular filtration rate, and high-sensitivity C-reactive protein. Data are shown as geometric means (grip strength), or mean (leg extension power) and 95% confidence interval.

In our study, grip strength differed significantly between participants with and those without hyperuricemia (geometric mean and 95% CI: 40.3 [39.2–41.3] kg vs. 41.9 [41.3–42.5] kg; *P* = 0.01) (Figure 1) after adjustment for age, BMI, smoking status, drinking frequency, education levels, occupation, physical activity, daily energy and protein intakes, metabolic syndrome, depressive symptoms, eGFR, and hs-CRP. Similar results were obtained when the leg extension power was compared, although the result was statistically not significant (*P* = 0.09).

**Figure 2:**
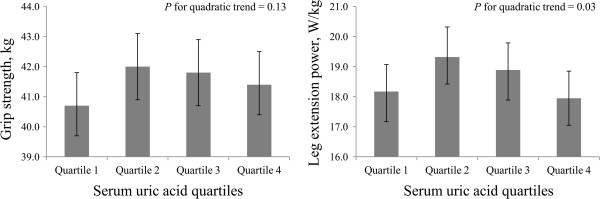
**Univariate inverted J-shaped association of quartiles of serum uric acid with muscle strength.** Data are shown as geometric means (grip strength), or mean (leg extension power) and 95% confidence interval.

Furthermore, we also investigated the association of serum UA quartiles with muscle strength by using univariate and multivariate analyses. In univariate analysis (Figure 2), serum UA levels showed an inverted J-shaped curve with grip strength (mean and 95% CI: Q1, 40.7 [39.7–41.8] kg; Q2, 42.0 [40.9–43.1] kg; Q3, 41.8 [40.7–42.9] kg; Q4, 41.5 [40.4–42.5] kg; *P* for quadratic trend = 0.13) and leg extension power (Q1, 18.2 [17.2–19.1] W/kg; Q2, 19.3 [18.4–20.3] W/kg; Q3, 18.9 [17.9–19.8] W/kg; Q4, 18.0 [17.0–18.9] W/kg; *P* for quadratic trend = 0.03). The inverted J-shaped association between UA quartiles and grip strength (*P* for quadratic trend: model 1 = 0.07, model 2 = 0.09, model 3 = 0.04, and model 4 = 0.05, respectively) and leg extension power (*P* for quadratic trend: model 1 = 0.06, model 2 = 0.05, model 3 = 0.03, and model 4 = 0.03, respectively) were unchanged even after adjustment for potential confounding factors, including age, BMI, smoking status, drinking frequency, education levels, occupation, physical activity, daily energy and protein intakes, metabolic syndrome, depressive symptoms, eGFR, and hs-CRP in the multivariate model (Table 3). To be specific, in model 4, we found a high value for muscle strength in the second UA quartile as compared with the first quartile, although the difference was not significant. Meanwhile, both grip strength and leg extension power were found to be significantly lower across the latter 3 quartiles of serum UA levels (*P* for linear trend = 0.05) (data not shown).

**Table 3:** Multivariate adjusted mean values (95% confidence interval) of muscle strength by serum UA quartiles

	**Serum UA quartiles, mg/dL**				***P ***^**1**^	***P ***^**2**^
	**<5.4**	**5.4–6.0**	**6.1–6.8**	**>6.8**		
Grip strength, kg *						
Participants (n = 586)	152	144	145	145	―	―
Model 1 ^3^	41.1 (40.1–42.1)	42.1 (41.1–43.2)	41.8 (40.8–42.8)	40.9 (39.9–42.0)	0.74	0.07
Model 2 ^4^	41.2 (40.2–42.3)	42.1 (41.0–43.1)	41.8 (40.8–42.9)	40.8 (39.8–41.9)	0.55	0.09
Model 3 ^5^	41.6 (40.6–42.7)	42.2 (41.1–43.2)	41.8 (40.8–42.9)	40.3 (39.2–41.3)	0.07	0.04
Model 4 ^6^	41.6 (40.6–42.6)	42.2 (41.2–43.2)	41.8 (40.8–42.8)	40.4 (39.3–41.4)	0.09	0.05
Leg extension power, W/kg						
Participants (n = 355)	92	90	85	88	―	―
Model 1	18.1 (17.2–18.9)	19.2 (18.3–20.0)	18.8 (18.0–19.7)	18.3 (17.4–19.2)	0.84	0.06
Model 2	18.0 (17.2–18.9)	19.2 (18.4–20.1)	18.8 (17.9–19.7)	18.2 (17.4–19.1)	0.95	0.05
Model 3	18.3 (17.5–19.1)	19.2 (18.4–20.1)	18.9 (18.0–19.7)	17.9 (17.0–18.8)	0.43	0.03
Model 4	18.3 (17.5–19.2)	19.2 (18.4–20.1)	18.9 (18.1–19.8)	17.9 (17.0–18.8)	0.40	0.03

^1^*P* for linear trend.

^2^*P* for quadratic trend.

^3^ Adjusted for age and BMI.

^4^ Same as model 1 + education levels, occupation, smoking status, drinking frequency, physical activity, daily energy intake, and daily protein intake.

^5^ Same as model 2 + metabolic syndrome, depressive symptoms, and eGFR.

^6^ Same as model 3 + hs-CRP.

* Variables have been log-transformed and expressed as geometric means (95% confidence interval).

*Abbreviations*: *BMI* Body mass index, *eGFR* Estimated glomerular filtration rate, *hs-CRP* High-sensitivity C-reactive protein.

## Discussion

Our population-based cross-sectional study has demonstrated that hyperuricemia is associated with poor muscle strength after adjustment for potential confounders in Japanese adult men aged 30–83 years. Furthermore, we have found a significant inverted J-shaped curve between serum UA quartiles and muscle strength.

To the best of our knowledge, this study shows for the first time that muscle strength was much lower in persons with hyperuricemia than in those without hyperuricemia. This study supports previous findings of low relative skeletal muscle mass in persons with hyperuricemia, which reported that participants with serum UA levels >8 mg/dL had 2 times the risk of sarcopenia (assessed on the basis of skeletal muscle mass) compared with those with <6 mg/dL after adjustment for other covariates in a population of 40 years old and older [29]. In addition, the observed inverted J-shaped associations of serum UA levels with muscle strength in our investigation were in accordance with another cross-sectional population study in which 966 elderly subjects were analyzed [16]. The study showed that participants in the middle serum UA quintile tended to have less disability in instrumental activities of daily living and better lower extremity function than those with higher or lower UA levels. Although these results are cross-sectional, keeping serum UA at an optimal level may contribute to maintaining skeletal muscle mass. It is interesting to note that most of the epidemiologic studies have indicated that serum UA levels showed a J-shaped association with cardiovascular events [11] and all-cause mortality [30,31], implying that both a low and a high UA level may lead to a higher risk of cardiovascular event or mortality.

On the other hand, a most recent prospective cohort study was the first to report a linear association of circulating UA levels with grip strength or/and leg extension power in elderly persons during a 3-year follow-up period [10]. Given its powerful antioxidant capacity, UA may protect skeletal muscle function from ROS-induced protein oxidative damage. However, the data in that study were unable to demonstrate the negative impact of high UA levels on muscle strength, as shown here. The different results between these 2 studies may be due to the disparity in age in the sample populations studied. It is logical to hypothesize that the antioxidant role of UA might be more relevant in older subjects than in younger subjects because the skeletal muscles in elderly persons have reduced overall antioxidant capacity to counteract ROS [32]. Moreover, another explanation is that the mean level (SD) of serum UA is 6.1 (1.2) mg/dL in this study, which is higher than the 5.0 (1.2) mg/dL (in both men and women) from the previous study. It is well known that an increased UA level is related to high inflammatory cytokines [14], a contributor to poor muscle strength [33].

In the current study, higher UA levels were associated with poor grip strength and leg extension power by the following possible mechanisms. First, elevated UA concentrations are related positively to systemic inflammation, particularly in the highest category of serum UA [14]. Indeed, urate crystals contribute to the inflammatory response through the release of pro-inflammatory mediators [34], and the risk of urate crystal formation/precipitation increases when the UA concentration exceeds 6.3 mg/dL [35]. As a prominent marker of systemic chronic inflammation, CRP has been associated with poor muscle strength [33,36,37]. Likewise, Cesari et al. [33] and Barbieri et al. [38] reported that the pro-inflammatory cytokine interleukin-6 (IL-6), which can promote CRP synthesis [39], is an independent predictor of poor muscle strength, especially in subjects with high IL-6 blood levels. In this study, however, the results of an inverted J-shaped curve between serum UA and muscle strength did not appreciably change after additional adjustment for hs-CRP, suggesting that inflammation is not the principal mechanism mediating this association among Japanese adult men. Second, the negative impact of high UA concentrations on muscle strength may be largely due to the serum UA-induced pro-oxidant capacity at higher than normal levels. The antioxidant compound UA may become pro-oxidant when concentrations reach hyperuricemic levels, and the surrounding oxidant milieu, acidity, or the depletion of other local antioxidants may regulate the antioxidant/pro-oxidant switch [17]. In fact, serum carbonylated protein and skin advanced glycation end products, which are widespread indicators of oxidative damage, have been associated with poor muscle strength in humans [40,41].

In addition, there is one hypothesis that may explain the lower muscle strength observed in the first UA quartile. A low serum UA level may reflect a decreased antioxidant capability, as UA was considered an important antioxidant in the blood [6-9]. Moreover, it is worth noting that adjustment for daily energy and protein intakes did not change the results of our study. These data seem to exclude the possibility of a role of malnutrition in the link, although low UA level may be a marker of low nutrient status [30], which was associated with poor grip strength [42].

Although this study has revealed important insights into the association between serum UA levels and muscle strength, it does have limitations. First, the association between serum UA and muscle strength is temporarily due to the cross-sectional nature of this study, and thus, a prospective study is necessary to confirm the causality of this association further. Second, we did not perform direct measurement of the UA antioxidant capacity as well as the total antioxidant capacity in the collected samples. Third, because we could not ascertain the site of inflammation in the present study, systemic or chronic inflammation may be present independent of serum UA. Last, the analysis was carried out only in men, and thus, our results cannot be directly generalized to women.

## Conclusions

Our cross-sectional study has, for the first time, revealed a negative impact of hyperuricemia on grip strength and leg extension power among Japanese men, after adjustment for additional covariates. Furthermore, the current study demonstrated that the association between serum UA levels (quartiles) and muscle strength does not show a linear trend but an inverted J-shaped curve. Future research should focus on clarifying the causality in association between circulating UA levels and muscle strength.

## Abbreviations

ANCOVA: Analysis of covariance; BDHQ: Brief self-administered diet history questionnaire; BMI: Body mass index; CI: Confidence interval; DNA: Deoxyribo nucleic acid; eGFR: Estimated glomerular filtration rate; HDL-C: High-density lipoprotein cholesterol; hs-CRP: High-sensitivity C-reactive protein; IL-6: Interleukin-6; MET: Metabolic equivalent; ROS: Reactive oxygen stress; SD: Standard deviation; UA: Uric acid.

## Competing interests

The authors declare that they have no competing interests.

## Authors’ contributions

CH, KN, and RN: study concept and design; CH, KN, YK, LG, HM, YC, MC, AO, HG, and HT: acquisition of subjects and data; CH, KN, LG, HM, YC and RN: analysis and interpretation of data; CH: drafting of the manuscript; YK, MC, AO, HG, and HT: administrative support; CH, KN, and RN: study supervision. All authors read and approved the final manuscript.

## Pre-publication history

The pre-publication history for this paper can be accessed here:

http://www.biomedcentral.com/1471-2474/14/258/prepub
